# Investigating the contribution of cytoarchitecture to diffusion MRI measures in gray matter using histology

**DOI:** 10.3389/fnimg.2022.947526

**Published:** 2022-09-13

**Authors:** Madhura Baxi, Suheyla Cetin-Karayumak, George Papadimitriou, Nikos Makris, Andre van der Kouwe, Bruce Jenkins, Tara L. Moore, Douglas L. Rosene, Marek Kubicki, Yogesh Rathi

**Affiliations:** ^1^Graduate Program for Neuroscience, Boston University, Boston, MA, United States; ^2^Psychiatry Neuroimaging Laboratory, Brigham and Women's Hospital, Harvard Medical School, Boston, MA, United States; ^3^Center for Morphometric Analysis, Massachusetts General Hospital, Charlestown, MA, United States; ^4^Martinos Center for Biomedical Imaging, Massachusetts General Hospital, Charlestown, MA, United States; ^5^Anatomy and Neurobiology, Boston University School of Medicine, Boston, MA, United States; ^6^Center for Systems Neuroscience, Boston, MA, United States

**Keywords:** diffusion MRI, histology, validation, gray matter, rhesus monkey, imaging biomarkers

## Abstract

Postmortem studies are currently considered a gold standard for investigating brain structure at the cellular level. To investigate cellular changes in the context of human development, aging, or disease treatment, non-invasive *in-vivo* imaging methods such as diffusion MRI (dMRI) are needed. However, dMRI measures are only indirect measures and require validation in gray matter (GM) in the context of their sensitivity to the underlying cytoarchitecture, which has been lacking. Therefore, in this study we conducted direct comparisons between *in-vivo* dMRI measures and histology acquired from the same four rhesus monkeys. Average and heterogeneity of fractional anisotropy and trace from diffusion tensor imaging and mean squared displacement (MSD) and return-to-origin-probability from biexponential model were calculated in nine cytoarchitectonically different GM regions using dMRI data. DMRI measures were compared with corresponding histology measures of regional average and heterogeneity in cell area density. Results show that both average and heterogeneity in trace and MSD measures are sensitive to the underlying cytoarchitecture (cell area density) and capture different aspects of cell composition and organization. Trace and MSD thus would prove valuable as non-invasive imaging biomarkers in future studies investigating GM cytoarchitectural changes related to development and aging as well as abnormal cellular pathologies in clinical studies.

## Introduction

Diffusion MRI is a widely used *in-vivo* imaging method that measures the displacement of water molecules within brain tissue. Since the water movement is affected directly by the underlying biology, dMRI can provide a non-invasive measure of the underlying tissue microstructure and offers a unique opportunity to conduct neuroimaging studies investigating microstructural brain changes. DMRI has been shown to be sensitive to changes in gray and white matter associated with brain development (Tanner et al., 1999; Yoshida et al., 2013), and aging (Giorgio et al., 2010; Lebel et al., 2012), as well as psychiatric and neurological disorders such as amyotrophic lateral sclerosis (ALS) (Baek et al., 2020), multiple sclerosis (MS) (Inglese and Bester, 2010), traumatic brain injury (TBI) (Laitinen et al., 2015; Zhang et al., 2017), Alzheimer's disease (Stebbins and Murphy, 2009), Parkinson's disease (Zhang and Burock, 2020), depression (Coloigner et al., 2019), and schizophrenia (Kubicki et al., 2007). Characterization of brain microstructure with this imaging method thus provides an opportunity to study developmental and aging related changes as well as disease related abnormalities.

However, dMRI can only provide indirect measures of the underlying microstructural properties of the tissue due to limited spatial resolution of the data. Further, dMRI provides an ensemble average of the displacement of water molecules within cell bodies, its processes (axons, dendrites), and extra-cellular spaces which are several orders of magnitude smaller than the millimeter scale image resolution. This necessitates the need for validation of dMRI measures. Currently, postmortem histological studies remain the gold standard to study tissue microstructure. So far, they have provided important information about gray matter (GM) microstructure at the cellular level, in healthy individuals as well as patients with psychiatric and neurological disorders (Huttenlocher, 1979, 1984; Rakic et al., 1986; Stockmeier and Rajkowska, 2004; Williams et al., 2013; Khaw et al., 2021). Therefore, validating the biological basis of these dMRI measures using postmortem tissue from animals scanned premortem can help establish the specificity and the interpretation of diffusion MRI studies in healthy and clinical populations as well as push the frontiers for the diagnosis and treatment of neurological and psychiatric disorders.

Several studies have been conducted in white matter for *ex-vivo* validation of dMRI models and the derived measures against relevant histological measures. These studies include those that validate dMRI tractography, fiber orientation, and other microstructural properties of axonal bundles estimated using various dMRI models, e.g., diffusion tensor imaging (DTI), high angular resolution diffusion-weighted imaging (HARDI), neurite orientation dispersion and density imaging (NODDI), and white matter tract integrity (WMTI) (Leergaard et al., 2010; Gao et al., 2013; Schilling et al., 2018; Gutierrez et al., 2020; Zhou et al., 2020; Leuze et al., 2021; Yendiki et al., 2021) with the relevant histological measures. Histological measures were computed in these dMRI validation studies, using various postmortem techniques such as neural tracer data (Gutierrez et al., 2020), myelin stains (Zhou et al., 2020), label-free optical imaging techniques (Yendiki et al., 2021), and optical imaging of fluorescently labeled neurofilaments and vasculature in 3D tissue cuboids cleared using the clear lipid-exchanged acrylamide-hybridized rigid imaging/immunostaining/*in situ*-hybridization-compatible tissue hydrogel (CLARITY) (Leuze et al., 2021). Animal models such as rats (Leergaard et al., 2010), marmosets (Gutierrez et al., 2020), squirrel monkeys (Schilling et al., 2018), macaques (Leuze et al., 2021) and humans (Zhou et al., 2020) were used to conduct these studies.

All of the above studies were primarily focused on white matter microstructure validation. In contrast, only a handful of studies have conducted postmortem validation of dMRI models and derived measures in GM (Kroenke et al., 2007; Bock et al., 2010; Jespersen et al., 2012; Laitinen et al., 2015; Seehaus et al., 2015; Khan et al., 2016; Dyrby et al., 2018; Shimony et al., 2018; Maiter et al., 2021; Salo et al., 2021). Moreover, the majority of these studies have used induced lesions (Laitinen et al., 2015), neonatal enucleation (Bock et al., 2010) or animal disease models of depression (Khan et al., 2016) or Parkinson's disease (Shimony et al., 2018) as a way of validation. Previous studies conducted in animal models of disorders have used histological techniques showing dMRI measures in GM regions to be sensitive to the underlying pathologies, e.g., neurodegeneration, myelinated fiber loss (Laitinen et al., 2015), and changes in neuronal density (Khan et al., 2016) and fiber density (Shimony et al., 2018). A few other histological validation studies conducted in normal, unaffected gray matter, reported a strong relationship between dMRI measures estimated from DTI, NODDI and constrained spherical deconvolution (CSD) models, and underlying properties of axonal, dendritic, and myelin microstructure in the neuropil (Kroenke et al., 2007; Jespersen et al., 2012; Seehaus et al., 2015; Maiter et al., 2021; Salo et al., 2021).

Even so, gray matter is comprised of not just dendrites and axonal bundles but also cell bodies and extracellular matrix. In GM, cellular composition and organization of cell bodies is very important to study because disorders such as schizophrenia, autism, bipolar disorder, and 22q11.2 deletion syndrome (22q11DS) have been shown to be associated with cytoarchitectural pathologies such as abnormal neuronal migration, changes in cell density, morphology, and other cytoarchitectural abnormalities (Arnold et al., 1991; Avino and Hutsler, 2010; Muraki and Tanigaki, 2015; Cho et al., 2016; Tee et al., 2017; Kikinis et al., 2019; Harrison et al., 2020). So far, there is only one model, the soma and neurite density MRI (SANDI) model, that has primarily focused on the characterization of cellular and neurite densities in gray matter (Palombo et al., 2020). Validation was provided for this model by comparing the SANDI-derived soma fraction in gray matter with the Allen mouse brain atlas contrast that represents the cellular density in the mouse brain (Ianuş et al., 2021). However, the SANDI model requires specific acquisition parameters (i.e., very high b-values) which can only be acquired on preclinical or specialized ultra-high gradient strength MRI scanners posing challenges for translation to human application (Palombo et al., 2020; Ianuş et al., 2021). Additionally, it is important to note that in most of the above-mentioned studies conducted in GM, postmortem validation was done using *ex-vivo* dMRI data. Imaging measures derived from the *ex-vivo* dMRI data cannot be directly translated to those measured *in-vivo*. The reason for this lies in the altered properties of the fixed postmortem tissue such as, significantly reduced intrinsic diffusivity and inhomogeneous shrinkage in brain volume due to formalin fixation across brain regions (Siegel et al., 1985; Rane and Duong, 2011; Lerch et al., 2012; Roebroeck et al., 2019). These challenges associated with using the *ex-vivo* dMRI data make it difficult to translate the results to *in-vivo* human studies.

A commonly used dMRI model, diffusion tensor imaging (DTI) (Alexander et al., 2007; O'Donnell and Westin, 2011) and a recently developed biexponential model (Rathi et al., 2013; Ning et al., 2015) have been used in several *in-vivo* human studies (especially the DTI model) to understand changes related to normal development, aging, and clinical conditions, showing strong translational value (Kubicki et al., 2007; Lebel et al., 2012; Yoshida et al., 2013; Avram et al., 2016; Wu et al., 2019; Baxi et al., 2020; Le et al., 2020). Measures derived from both these models individually have shown the ability to capture the influence of both genetic and environmental factors on brain microstructure in human subjects (Elman et al., 2017; Vuoksimaa et al., 2017; Gustavson et al., 2019; Baxi et al., 2020). Thus, DTI and biexponential model derived measures once validated, have the potential to be individually used as imaging biomarkers to study disease-related as well as normal development and aging-related brain changes.

The DTI model is by far the simplest and most commonly used model for dMRI data to study brain microstructure in health and disease. The commonly used scalar measures derived from this model are fractional anisotropy (FA) and trace that measure biophysical properties of the underlying tissue. FA measures the degree of anisotropy in the water diffusion within a voxel and trace describes the average mobility of water molecules in the tissue. However, the DTI model relies on the assumption of anisotropic Gaussian diffusion of water molecules in the tissue (Basser et al., 1994; Jones and Cercignani, 2010; O'Donnell and Westin, 2011). In contrast, the biexponential model allows modeling of non-Gaussian water diffusion behavior in complex brain tissue thus making it a more realistic model to probe the complex tissue microstructure (Özarslan et al., 2013; Rathi et al., 2013; Ning et al., 2015). The diffusion propagator derived from the biexponential model uses a mixture of Gaussians (Rathi et al., 2013) to represent the diffusion signal across multiple b-values. In this case, all derived measures have simple analytical expressions, making them easier and more robust to estimate. The scalar measures derived from this biexponential model are mean-squared-displacement (MSD) and return-to-origin-probability (RTOP). MSD primarily captures the displacement of fast-moving water molecules while RTOP measures the probability of a water molecule returning to its starting position in a given experimental diffusion time and is an indicator of restricted diffusion (Assaf et al., 2002; Wu and Alexander, 2007; Özarslan et al., 2013; Ning et al., 2015; Boscolo Galazzo et al., 2018; Afzali et al., 2021). For example, if water is highly restricted, one might expect higher RTOP as there is a higher likelihood of the water molecules coming back to their starting positions. On the other hand, in the ventricles where there is little restriction, the RTOP is low. Similarly, MSD will be high in the ventricles and lower in restricted regions.

However, the question of how the fundamental GM microstructural properties such as cell size, cell packing density, spatial arrangement, and geometry of cell bodies influence these measures (FA, Trace, MSD and RTOP) derived from these two *in-vivo* dMRI models (DTI and Biexponential), still remains unclear. Quantifying the connection between these dMRI measures and histological properties of cellular architecture is critical for progress in the field and is the main focus of this work.

Therefore, in this study, we investigated how the underlying cytoarchitecture in gray matter is related to DTI and biexponential measures. To validate these diffusion measures (trace, FA, MSD and RTOP), we compared them with the histology based cytoarchitectural regional tissue properties of average and heterogeneity of cell area density. Specifically, we used digitized Nissl-stained slices and *in-vivo* high-resolution multi-shell dMRI data acquired from the same four monkeys to quantify the contribution of underlying cytoarchitecture to the dMRI measures.

## Methods

### Demographics

This study included *in-vivo* MRI scans and archived brain tissue from four rhesus monkeys (all male) 19–20 years old (equivalent to 57–60 years in humans; Tigges et al., 1988). A small age range was chosen specifically to reduce age effect on variability across the histological and imaging measures. Health records of these monkeys were screened to exclude any possible confounding clinical diseases or experimental manipulations that could impact normal aging.

Monkeys were obtained from national primate research facilities or private vendors and had known birth dates and complete health records. Monkeys were housed in the Animal Science Center of Boston University School of Medicine which is AAALAC accredited. All procedures were approved by the Boston University Institutional Animal Care and Use Committee and complied with the NIH guidelines for the care and use of laboratory animals.

### Structural and diffusion MRI

#### MRI acquisition

Structural and diffusion MRI scans were acquired using the same protocol and scanner for all four monkeys as described below. Monkeys were anesthetized with ketamine (10 mg/kg) and xylazine (0.10 mg/kg) and placed into a 3 Tesla Connectome MRI scanner (Siemens), located at the Martinos Center for Biomedical Imaging using an MRI compatible stereotactic frame that fixed the monkey head in the standard coronal plane (Saunders et al., 1990). The following parameters were used for acquiring structural (sMRI) and dMRI scans, which took around 3 h:

*T1 MPRAGE Structural MRI:* TI/TR/TE = 1100/2530/1.37 ms, flip angle = 7°, FOV = 160 × 160 mm^2^, slices = 176, voxel size = 0.6 × 0.6 × 0.59 mm^3^, bandwidth = 650 Hz/pixel, 32 channel head coil, and acceleration factor of 2.

*Multi-shell Diffusion-weighted MRI*: Spin-echo single-shot EPI sequence, TR/TE = 15700/73 ms, flip angle = 90°, slices = 118, FOV = 140 × 140 mm^2^, voxel size = 0.8 × 0.8 × 0.8 mm^3^, bandwidth = 1185 Hz/pixel. Three repetitions of diffusion MRI scans were acquired with 11 b-value of 0 s/mm^2^ and 60 directions for each b-value of 1,000, 2,000, and 3,000 s/mm^2^.

#### MRI preprocessing

**Figure 1:**
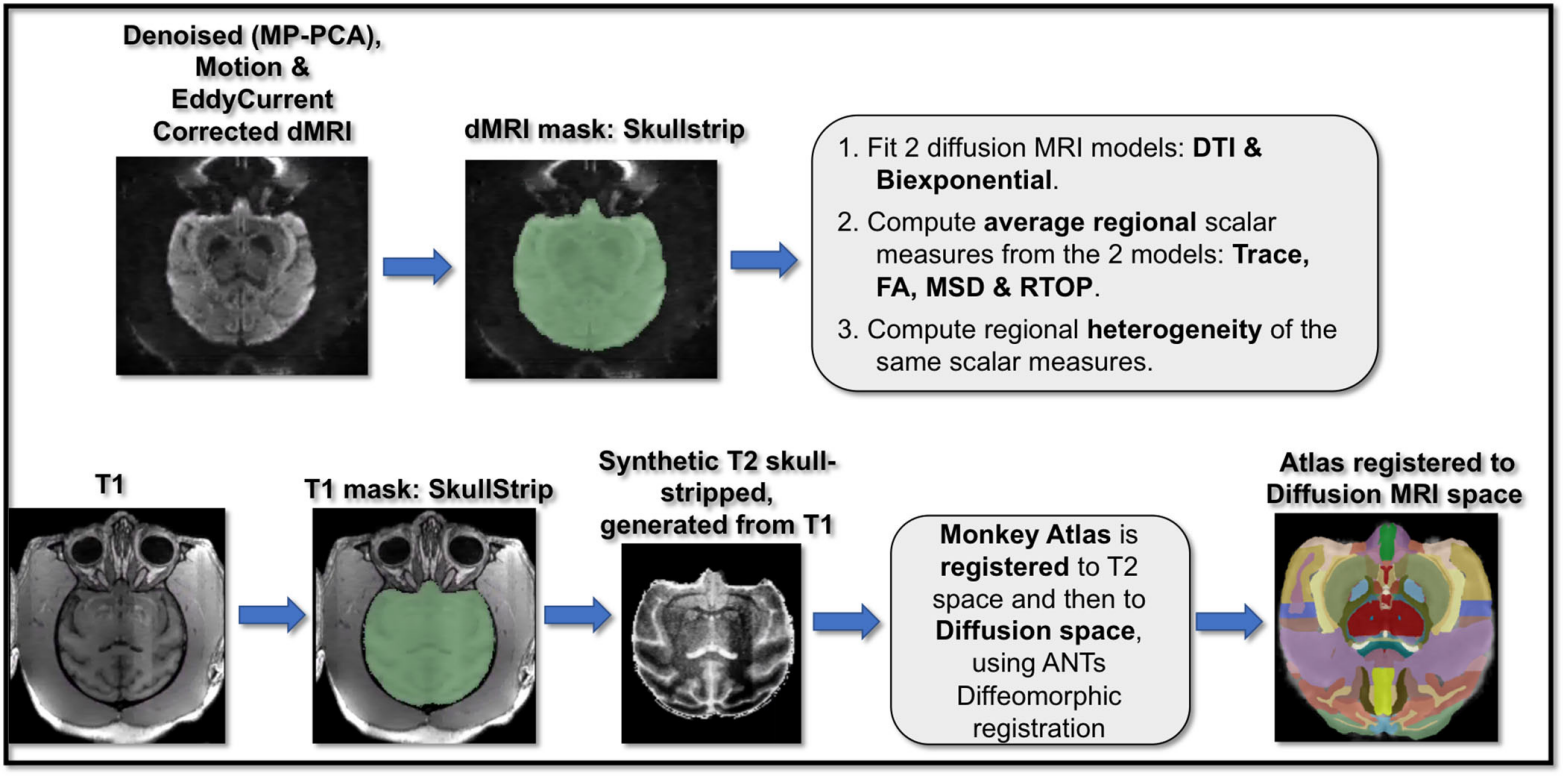
Pipeline for dMRI and T1-weighted scans preprocessing and analysis. dMRI data underwent a cascade of preprocessing steps including MP-PCA denoising, motion correction, and eddy current correction, followed by masking and brain extraction. T1-weighted images also underwent preprocessing steps including bias field correction and brain extraction. Next, brain macaque anatomical atlas was registered to T1 space and then to diffusion space. Two dMRI models of DTI and the biexponential model were fit to the dMRI data and corresponding scalar maps of trace, FA (from DTI) and MSD, RTOP (from biexponential model) were computed. Using the dMRI scalar maps computed from the two model fits and the atlas registered to the dMRI data, regional average and variance/heterogeneity of these dMRI measures (FA, Trace, MSD and RTOP) were computed.

MRI scans were preprocessed using the standard in-house pipeline (https://github.com/pnlbwh) (Figure 1). Both sMRI and dMRI image modalities were visually inspected for significant artifacts and signal dropouts. Upon manual visual inspection of dMRI data, we observed that one of the three repetition scans from two monkeys, each had significant motion artifacts through most volumes and hence if one or more slices had motion artifacts the corresponding dMRI volume was removed from further processing. No data was removed from the remaining repetition scans as we were able to correct for minor motion artifacts. This data can be made available upon request.

*Diffusion-weighted MRI scans*: Diffusion MRI scans for each monkey included three repetitions/runs which went through the following preprocessing steps (Figure 1) that included MP-PCA denoising (Veraart et al., 2016) using https://github.com/NYU-DiffusionMRI/mppca_denoise code, followed by motion correction, brain extraction and eddy current correction using Functional Magnetic Resonance Imaging of the Brain (FMRIB) Software Library (FSL) software (http://www.fmrib.ox.ac.uk/fsl) (Smith et al., 2004) on each run. The three preprocessed runs were then averaged into one volume for each monkey.

*T1-weighted scans*: T1-weighted scans went through the preprocessing steps (Figure 1) that included skull stripping and bias field correction using FSL (Smith, 2002). Next, T2-weighted images were synthetically generated using in-house MATLAB scripts that performed a cascade of image processing steps including intensity reversal, log transformation for image enhancement and histogram equalization on preprocessed T1-weighted scans (Kubicki et al., 2019). T2-weighted images were generated for accurate co-registration of the T1-weighted images and associated brain parcellation with dMRI data (as the intensities of T2-weighted and b = 0 dMRI images are quite similar). In order to calculate regional diffusion MRI measures, a brain macaque atlas (Dubach and Bowden, 2009; Rohlfing et al., 2012) was non-linearly registered first to the T2-weighted images and then to the diffusion space for each monkey, using Advanced Normalization Tools (ANTs) registration (Avants et al., 2014).

#### Fitting diffusion MRI models

*DTI Model*: Conventional diffusion tensor imaging (DTI) model was fit using Slicer v4.8 (http://www.slicer.org), (Fedorov et al., 2012) to diffusion MRI data using the b = 0 and b = 1000 s/mm^2^ shells of the multi-shell diffusion MRI scans. DTI model is a single tensor model and relies on the assumption that water diffusion in the brain follows a mono-exponential Gaussian distribution (Basser et al., 1994; Jones and Cercignani, 2010). Using this DTI model, scalar maps of fractional anisotropy (FA) and trace (Tr) were computed for each monkey. FA represents the degree of anisotropy of the biological tissue and has been commonly used to study white matter structure. Trace describes the average mobility of water molecules in the tissue.

*Biexponential model*: A more sensitive model, i.e., biexponential model was fit to the multi-shell dMRI data, which allows modeling of non-Gaussian water diffusion behavior in brain tissue exhibited by the underlying biological structures e.g., myelin, cell bodies and its processes (Özarslan et al., 2013; Rathi et al., 2013; Baxi et al., 2020). The model consisted of a weighted mixture of two exponentials oriented in the same direction modeling the signal across multiple b-values and gradient directions (Mulkern et al., 1999; Rathi et al., 2013). CSF contamination was accounted for and removed when we modeled the diffusion behavior using the biexponential model. This model consisted of an isotropic compartment to model the CSF contamination apart from the restricted compartment to model the non-gaussian diffusion in the GM tissue for each voxel. This allowed us to remove CSF contamination before computing the dMRI measures used in further analysis. More details on this model can be found in a recently published paper by Baxi et al. (2020). Scalar maps of return-to-origin-probability (RTOP) and mean squared displacement (MSD) were then computed for each monkey using this biexponential model (Ning et al., 2015; Baxi et al., 2020). MSD primarily captures the displacement of fast-moving water molecules (i.e., it is more sensitive to larger displacements).

### Immunohistochemistry

#### Macaque postmortem tissue processing and histology

The four monkeys that were scanned *in-vivo* to acquire dMRI scans, were sacrificed shortly after the MRI scans. They were deeply anesthetized with sodium pentobarbital and perfused transcardially through the aorta with Krebs Henseleit buffer, pH 7.4 at 4°C for 5 min while fresh tissue samples were harvested. This was followed by perfusion with 4% buffered paraformaldehyde (pH 7.4, 37°C) for 10 min. Next, the brain was blocked, *in situ*, in the coronal stereotactic plane, removed from the skull and placed in the same paraformaldehyde fixative overnight at 4°C. It was then cryoprotected by incubations in 0.1 M phosphate buffer containing first 10% glycerol with 2% DMSO and then 20% glycerol and 2% DMSO (Rosene et al., 1986). Next it was flash frozen in isopentane at −75°C and stored at −80°C. Frozen sections were cut into interrupted series on a sliding microtome. Eight sections were cut at 30 μm thickness and one at 60 μm and this was repeated until the entire whole hemisphere brain was cut. As a result, sections within each series are spaced at 300 μm intervals. The 60 μm sections were mounted onto gelatin-albumin subbed microscope slides, dried overnight at room temperature and then stained with thionin to reveal cell bodies. This series was prepared for digitization as done here and for stereology which is more efficient with the thicker sections. The other 8 series of 30 μm sections are collected in phosphate buffer with 15% buffered glycerol and stored at −80°C until needed for other staining such as immunohistochemistry (IHC) (see Estrada et al. (2017) for examples). No 30 μm IHC series were analyzed for this study.

#### Microscopy digitization

We then digitized the stained slices by imaging them using a Zeiss Axioscan microscope with 10x objective to obtain Z-stacked images which were then merged into a single image per slice with resolution of 0.44 × 0.44 μm^2^. Z-stack imaging of tissue samples, allows for accurate visualization of the cell bodies in the tissue.

#### Cell segmentation of digitized histological slices

**Figure 2:**
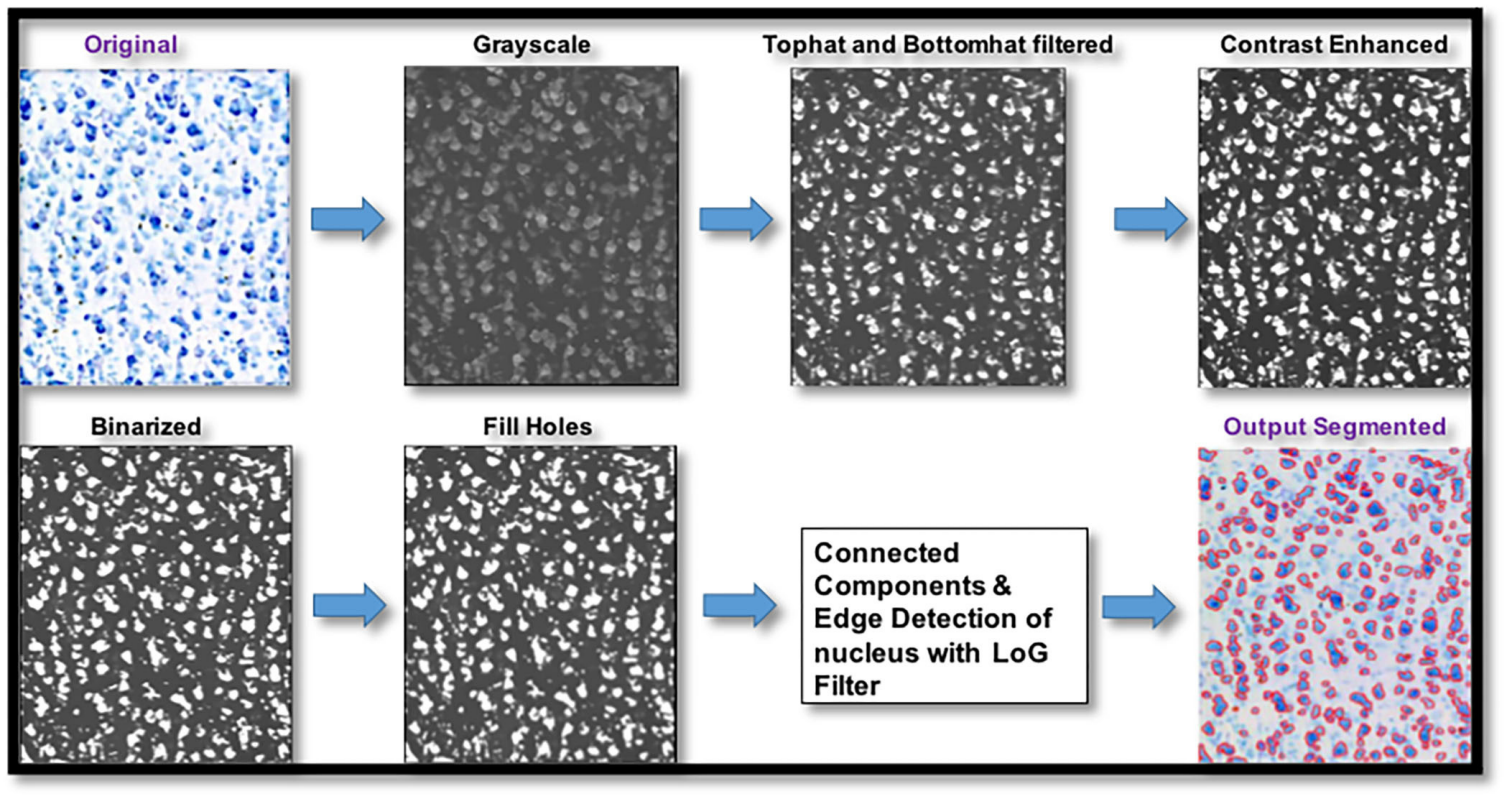
Pipeline for digitized histological sections preprocessing and cell segmentation. Digitized histological section images underwent preprocessing steps that involved top hat and bottom hat filtering, contrast enhancement, binarizing the image using a histogram-based threshold, filling holes followed by connected-component labeling for cell segmentation and edge detection with Laplacian of gaussian filter for plotting the edges of the cell bodies.

To avoid registration errors between histology and dMRI, we used manual delineations of nine cortical and subcortical regions on histology sections. We then segmented the histological slices to identify stained cell bodies using the MATLAB Image Processing Toolbox and Nuclei Counter Code (https://www.mathworks.com/matlabcentral/fileexchange/45174-nuclei-counter) (Figure 2). The segmentation quality of our code was assessed by comparing the automatically segmented samples with the manually segmented samples (performed by trained expert). Three samples were chosen randomly from the anterior, middle, and posterior sections of each ROI. Measures of Dice coefficient and error in cell area density were computed for each of these samples upon comparing the automated cell segmentation with the manual cell segmentation.

### Computing average and heterogeneity regional measures

**Figure 3:**
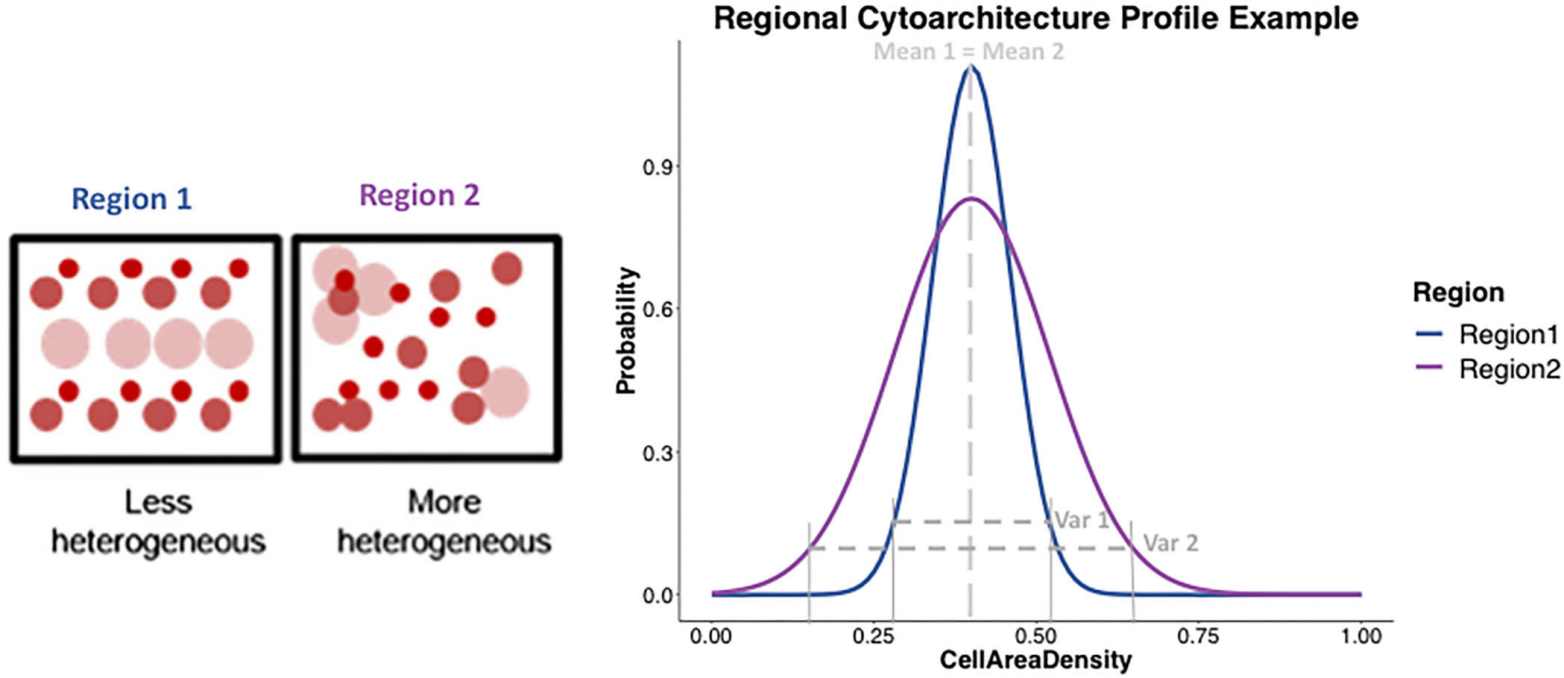
Example representation of the cytoarchitecture profile in different gray matter regions. (Left) Two boxes represent two regions, with circles representing cell bodies of different types, sizes, and uniformly or non-uniformly packed, showing region 1 (blue) being less heterogeneous and region 2 (purple) being more heterogeneous in the underlying cytoarchitecture. (Right) Plot shows an example of what the cytoarchitecture profile would be expected to look like for region 1 (blue) and region 2 (purple) using two different shaped distributions. Region 1 being less heterogeneous, would be expected to show a distribution profile (in blue) that has a certain mean and a tighter variance. On the other hand, a more heterogeneous region 2 would be expected to show a distribution profile (in purple) with similar mean but a much larger variance than region 2. The shape of the two-distribution profile for region 1 and region 2 can be seen to be described by both mean and the variance.

The distribution profile of diffusion measures and histological cytoarchitectural properties of cell composition in any brain region should be described by two metrics, mean/average and variance/heterogeneity, as opposed to just the mean/average which is commonly done. This is because two very differently shaped distribution profiles as seen in Figure 3 could have similar means and using only the mean to characterize a distribution profile would give the false impression of the distributions being similar when they might be different in their variance.

Since cortex is highly plastic and undergoes changes in cellular composition with age, this could alter the spatial organization of GM within a voxel and hence change heterogeneity measures (Zatorre et al., 2012; Rathi et al., 2014). The importance of using variance/heterogeneity to study the structure has also been shown by previous studies to differentiate between patients with early course schizophrenia and healthy controls (Seitz et al., 2018) and to study/detect disruption of migration patterns of neural crest cells in 22q11DS (Kikinis et al., 2019).

**Figure 4:**
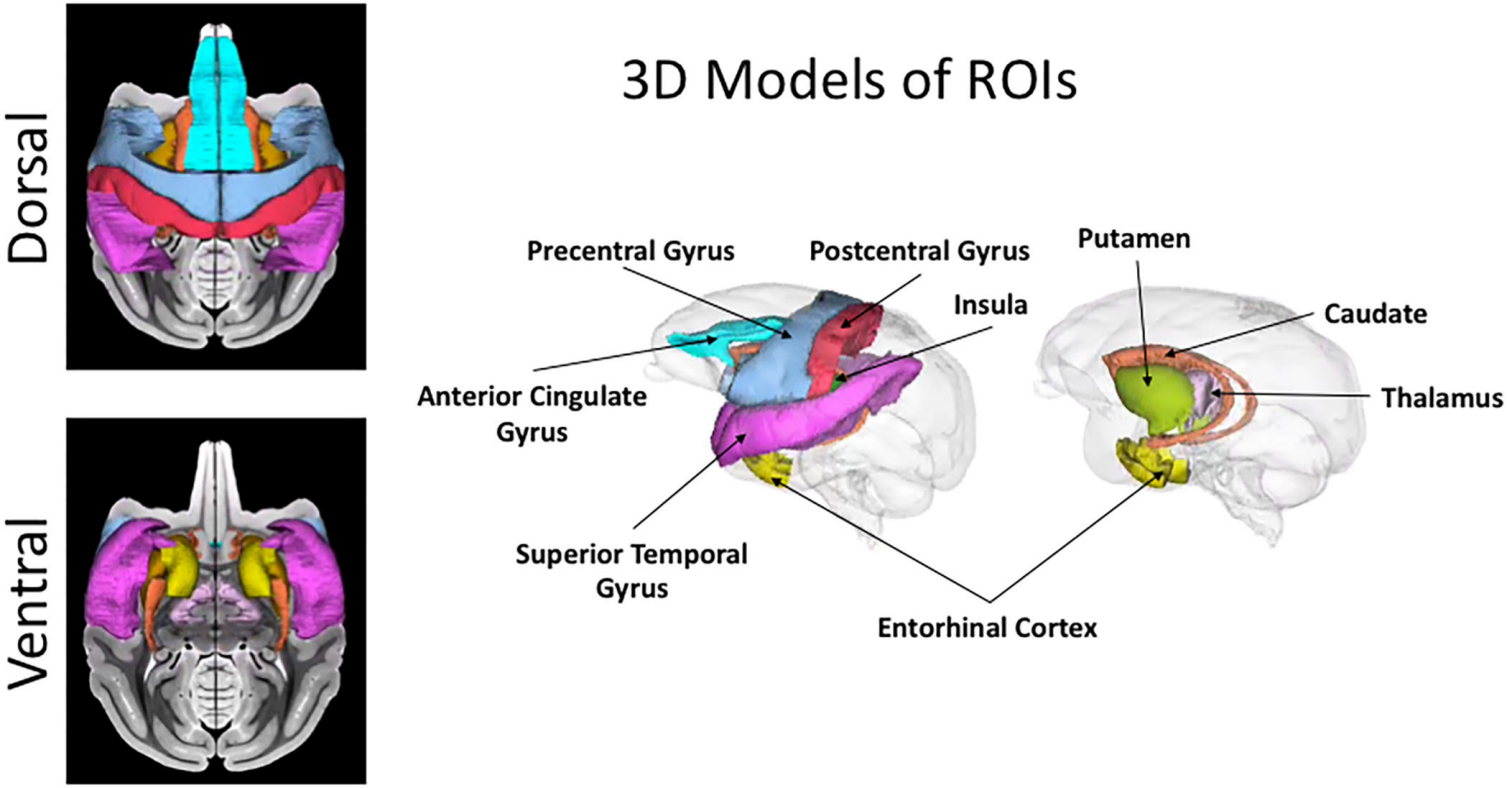
Cortical and subcortical ROIs. 3D rendering and projection on T1-weighted image of the **nine** regions used in this study for the region-based comparison between histology and dMRI are shown in this figure. The color coding for the regions is as follows: anterior cingulate gyrus (ACG) in sky blue, caudate in orange, entorhinal gyrus in yellow, insula in dark green, postcentral gyrus in red, precentral gyrus in light blue, putamen in light green, superior temporal gyrus (STG) in dark pink, and thalamus in light pink.

Hence, in this study, we computed both mean/average and variance/heterogeneity of dMRI and histological measures for nine gray matter regions. It is well known from neurobiology literature (Brodmann, 1909; Vogt and Vogt, 1926; Goldman-Rakic, 1982) that different brain areas including cortical and subcortical regions, vary in their cellular composition and organization across different gray matter regions. The nine GM ROIs used in this study (Figure 4), were chosen to get a good sampling of different cytoarchitectural properties. Hence it included cortical regions of anterior cingulate gyrus (ACG), precentral gyrus, postcentral gyrus, superior temporal gyrus (STG), insular cortex and entorhinal cortex along with subcortical regions of caudate, putamen, and thalamus.

### Estimating regional average and heterogeneity DMRI measures

The brain macaque atlas (Dubach and Bowden, 2009; Rohlfing et al., 2012) was registered to the dMRI scans for each monkey which was used to compute regional average (avgdMRImeas) and heterogeneity (hdMRImeas) of dMRI measures for nine GM regions of interest (ROIs). These measures were computed using FA, trace, MSD and RTOP for each monkey. AvgdMRImeas was computed by taking the average of a given dMRI measure (FA/trace/MSD/RTOP) over all voxels in an ROI. HdMRImeas is defined as the inter-voxel variance in a given dMRI measure (FA/trace/MSD/RTOP) across a region.


$$avgdMRImeas\;=\;\frac{1}{N}\times \sum_{i=1}^{N}dMRImeas_{i}$$

N is the number of voxels in the ROI and the dMRI measure in a voxel is indexed by i.


$$hdMRImeas\;=\;\frac{1}{N^{2}}\times \sum_{i=1}^{N}\sum_{j=1}^{N}∥dMRImeas_{i}-dMRImeas_{j}∥$$

N is the number of voxels in an ROI and the dMRI measure in a voxel is indexed by i or j.

### Estimating regional average and heterogeneity histological measures

**Figure 5:**
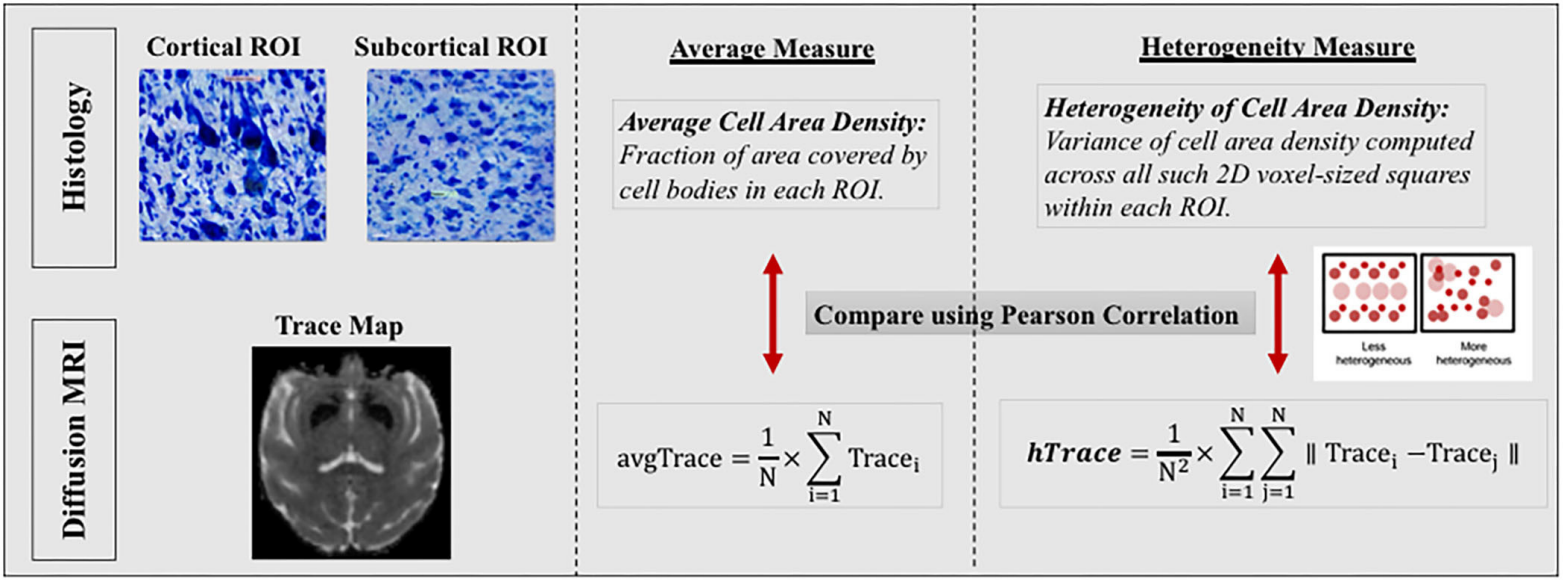
Comparison between regional histology and dMRI measures. In this figure, we only show the comparison between histology and dMRI measure of trace as an example. Similar comparisons were preformed between histology and other dMRI measures of FA, MSD and RTOP. (Left section) Top histology images are zoomed in sections, from a cortical region of precentral gyrus showing large Betz cells and heterogeneous cytoarchitecture, and another from a subcortical region of thalamus showing homogeneous cytoarchitecture. Bottom dMRI image is a trace map from the same monkey as the top histology images. (Middle and Right sections) Regional average and variance/heterogeneity measures were computed for the same nine regions from the histology and dMRI data which were then compared using Pearson correlation. Regional average and heterogeneity of cell area density were computed from the histology data to be compared with corresponding regional average and heterogeneity of dMRI measures (FA, trace, MSD and RTOP). Right section also shows a small graphical representation of how a less heterogeneous (left box) vs. a more heterogeneous region (right box) would look like, using circles to represent cell bodies of different cell sizes, types, and locations.

Next, we computed analogous histological measures of cell area density and heterogeneity of cell area density for the same 9 ROIs for each monkey, to compare with regional average and heterogeneity of dMRI measures (Figure 5). Cell Area Density for each region was computed as the fraction of area covered by cell bodies in each ROI. In order to compute the heterogeneity of cell area density, each ROI was resampled into 2D grid to match the diffusion voxel size. Variance of cell area density was then computed across all such 2D voxel-sized squares within each ROI, which provided us the heterogeneity in cell area density.

### Comparing *in-vivo* DMRI measures with *ex-vivo* histology measures

In order to quantify the contribution of regional histological measures to the dMRI measures (FA/trace/MSD/RTOP), we conducted Pearson correlations between histology-derived and dMRI-derived measures (Figure 5). We performed correlations between average histology and dMRI measures separately in cortical and subcortical regions, given the major cytoarchitectural differences known to exist between cortical and subcortical regions (Amunts and Zilles, 2015). All *p*-values were corrected for multiple comparisons using FDR-correction. We also checked for the effect of regional volume on regional average cell area density and did not find any significant effect.

## Results

### Cell segmentation algorithm quality assessment

Quality of our automated cell segmentation was assessed by comparing the automatically segmented samples with the manually segmented samples that were chosen from the anterior, middle, and posterior sections of each ROI. Quantitative measures of dice overlap coefficient and error in cell area density were computed for each sample. Dice coefficient provides the similarity index between two segmentations and ranges from 0 to 1, with 1 signifying an absolute match. Samples from all three portions of all the ROIs showed high dice coefficient which varied between 0.91 and 0.99, and an average error in the estimated cell area density measure of less than 0.04.

### Comparing DMRI (*in-vivo*) measures with histology (*ex-vivo*) measures

Comparison between dMRI measures (DTI and biexponential model) and corresponding histology measures of cell area density computed for 9 GM regions from four rhesus macaques, showed the following results.

#### Average regional measures

**Figure 6:**
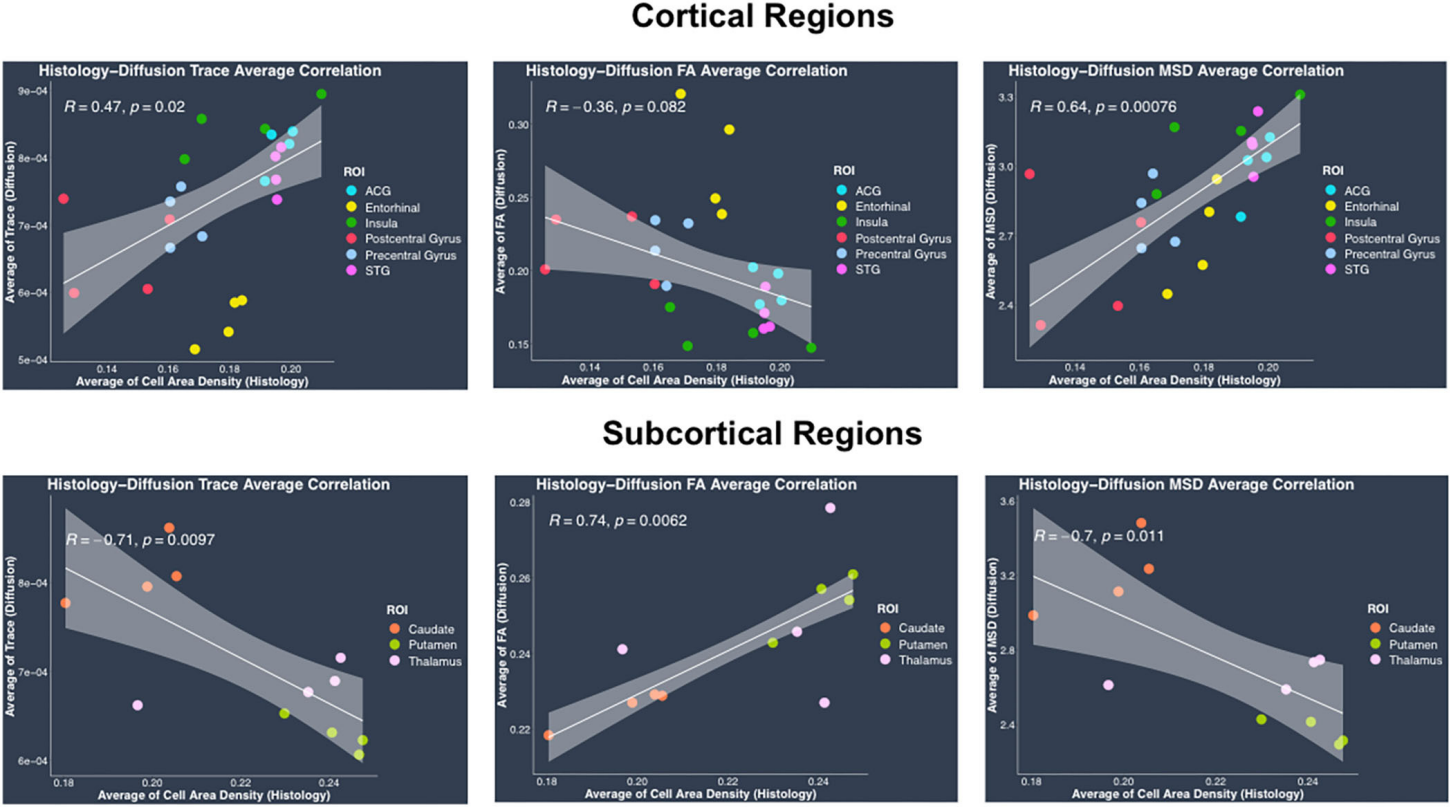
Average histology vs. diffusion MRI correlations separately in cortical and subcortical regions. (Top row) Plots show linear fits used to model the relationship between regional average of cell area density from histology and dMRI regional average measures of trace, FA and MSD in cortical regions, acquired from the same four monkeys. (Bottom row) Plots show linear regression fits for regional average cell area density (histology) vs. regional average dMRI measures of trace, FA and MSD in subcortical regions, acquired from the same four monkeys. All plots show 95% confidence band in gray color around the linear regression line. Pearson correlation co-efficient *r* and its *p*-value have been indicated on the top left corner for each plot. In each plot, individual points have been color coded to represent a region from the four monkeys. The color coding for the regions is as follows: anterior cingulate gyrus (ACG) in sky blue, caudate in orange, entorhinal gyrus in yellow, insula in dark green, postcentral gyrus in red, precentral gyrus in light blue, putamen in light green, superior temporal gyrus (STG) in dark pink, and thalamus in light pink.

Average cell area density values which were computed as the fraction of area covered by all cell bodies (Figure 6) were overall consistent with the previous studies (Semendeferi et al., 2001; Casanova et al., 2002) that measured Gray Level Index (GLI) which is defined as the fraction of the area covered by Nissl-stained neurons and glial cells in postmortem samples. Gray level index is thus the same as the average cell area density defined in this study. Average trace showed significant positive correlation with average cell area density in cortical regions (*r* = 0.47, *p* = 0.02), whereas it showed significant negative correlation in subcortical regions (*r* = −0.71, *p* = 0.0097) (Figure 6). Similarly, between average cell area density and average MSD, results showed a positive correlation in cortical regions (*r* = 0.64, *p* = 0.00076), whereas a negative correlation was shown in subcortical regions (*r* = −0.7, *p* = 0.011) (Figure 6). Average FA when correlated with average cell area density did not show significant correlation in cortical regions (*r* = −0.36, *p* = 0.082) but showed positive correlation in subcortical regions (*r* = 0.74, *p* = 0.0062) (Figure 6). Average RTOP did not show significant correlation (*p* > 0.05) with average cell area density in either cortical or subcortical regions. Variability in the histology and dMRI measures within each region can be observed across the four monkeys (same colors on Figure 6) showing the sensitivity to inter-subject differences in the brain structure. Even though measures for each region from all four monkeys show some variation, they tend to cluster together, highlighting the robustness of our results across monkeys.

#### Heterogeneity regional measures

**Figure 7:**
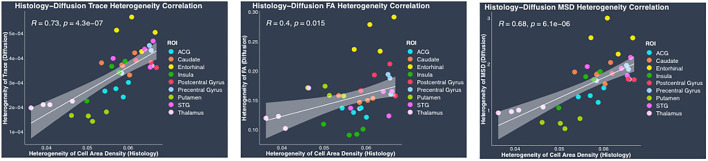
Heterogeneity in histology vs. diffusion MRI correlations. Plots show linear fits used to model the relationship between regional heterogeneity of cell area density from histology and dMRI regional heterogeneity measures of trace, FA and MSD acquired from the same four monkeys. All plots show 95% confidence band in gray color around the linear regression line. Pearson correlation co-efficient *r* and its *p*-value have been indicated on the top left corner for each plot. In each plot, individual points have been color coded to represent a region from the four monkeys. The color coding for the regions is as follows: anterior cingulate gyrus (ACG) in sky blue, caudate in orange, entorhinal gyrus in yellow, insula in dark green, postcentral gyrus in red, precentral gyrus in light blue, putamen in light green, superior temporal gyrus (STG) in dark pink, and thalamus in light pink.

Regional heterogeneity in cell area density measure computed from histology data showed a high positive significant correlation with DTI-derived hTrace (*r* = 0.73, *p* = 4.3^*^10^−7^) and biexponential model-derived hMSD (*r* = 0.68, *p* = 6.1^*^10^−6^) measures and moderate correlation with DTI-derived hFA (r = 0.4, *p* = 0.015) (Figure 7). No significant correlation was observed between the histological measure of regional heterogeneity in cell area density measure and biexponential model-derived hRTOP (*p* > 0.05) (Figure 7). We also performed correlations between heterogeneity histology and dMRI measures separately in cortical and subcortical regions, which showed similar trends as the results upon combining cortical and subcortical regions in the same analysis. Overall, these results showed that the higher the heterogeneity in cell area density, the higher the heterogeneity in trace and MSD (and FA to a smaller degree).

## Discussion

This study presents an important step toward quantifying the contribution of GM cytoarchitecture to *in-vivo* dMRI measures using histology in rhesus monkeys. This research paves the way for bridging the gap between the two distinct modalities and allows for a better and more informed use of dMRI in the future. Specifically, we aimed to investigate the influence of the underlying cell composition and spatial organization/distribution on dMRI measures estimated using the conventional DTI model (FA and trace) and an advanced biexponential model (MSD and RTOP). This was achieved by conducting correlations between regional average and heterogeneity of cell area density (estimated using Nissl-stained digitized histology data) and corresponding dMRI measures (FA, trace, MSD and RTOP), in the same four rhesus monkeys. The work presented here is based on *in-vivo* dMRI in non-human primate animal models and provides an important guide to interpreting the results of *in-vivo* dMRI measures in the human brain (Gao et al., 2013). The main finding of this study is that regional heterogeneity in trace and MSD measures from dMRI capture the underlying biological features of spatial arrangement of cell bodies as measured by heterogeneity of cell area density in that region.

Our results showed a statistically significant positive correlation between the histological measure of regional heterogeneity in cell area density and dMRI heterogeneity measures of trace and MSD. This suggests that the dMRI heterogeneity measures are sensitive to heterogeneity in the cell area density, driven by the variability in cell size and cell packing density in a region. A region is considered to be homogeneous in cytoarchitecture if the variation in the cell size and type is low and they are uniformly distributed across the region. Conversely, a region with cell bodies of varied sizes and shapes organized in a complex, non-uniform arrangement is considered to have a heterogeneous cytoarchitecture. Our findings show that hTrace and hMSD can capture the variability in the underlying cell composition and spatial arrangement.

GM regions are known to vary in their underlying cytoarchitecture, with some regions exhibiting more heterogeneity in the organization of cell bodies and its processes compared to others. Our results reflect these differences between regions. For example, the entorhinal cortex shows the highest heterogeneity in trace and MSD and also in heterogeneity in cell area density, as compared to their cortical and subcortical regions. This finding corroborates with previous postmortem studies reporting the complex cytoarchitectonic organization of the entorhinal cortex, which can be further differentiated into seven subfields on the basis of differences observed in the morphological features visible in Nissl- and fiber-stained preparations (Amaral et al., 1987; Sewards and Sewards, 2003). These studies found that rostrally located fields show a number of morphological inhomogeneities with neurons organized in patches in contrast to the caudally located subfields that more closely resemble the neocortex with neurons arranged in discrete radial columns (Amaral et al., 1987; Sewards and Sewards, 2003; de Góis Morais et al., 2020). Heterogeneity in cell area density observed in precentral gyrus and postcentral gyrus can be attributed to the presence of large pyramidal cells (Betz cells), in the layer V of precentral gyrus as well as somatotopic organization (Cusick et al., 1989; Krubitzer and Kaas, 1990; Fink et al., 1997; Eickhoff et al., 2007). Among the subcortical regions, our results show that caudate shows higher heterogeneity in cytoarchitecture as compared with putamen, which is consistent with a previous study that reported homogeneous cellular appearance of the putamen compared to the more prominent complex cellular islands consisting of densely packed neurons of variable cell sizes and shapes seen in the caudate nucleus (Goldman-Rakic, 1982).

Further, our results demonstrate that the dMRI measures of hTrace, hFA, hMSD and hRTOP show differences in their ability to capture the biological features of the underlying cytoarchitecture. Both hMSD and hTrace show similar high positive correlation with the histology measure of heterogeneity in cell area density. This could be explained by the similarities in the biophysical properties captured by these two measures derived from DTI and biexponential models. Previous studies have shown that MSD and trace measures are related *via* the Einstein diffusion equation (Wu and Alexander, 2007; Hosseinbor et al., 2013; Boscolo Galazzo et al., 2018) and have been reported to show visually correlated behavior with very similar tissue contrast maps (Wu and Alexander, 2007) and similar patterns when studied in patients with ischemic oedema (Alexander et al., 2007). These studies are thus consistent with our finding of similar patterns seen in the correlations between both hMSD and hTrace with heterogeneity in cell area density. However it is important to note that previous studies have also found MSD to be more sensitive to underlying microstructure as compared to trace, demonstrating the advantage of using the biexponential model measure of MSD (Boscolo Galazzo et al., 2018). In contrast to the findings from MSD and trace, hFA showed only low to moderate influence of heterogeneity in cell area density. FA is known to measure the restriction and anisotropy of water diffusion and, in WM it has been shown to capture the underlying biological properties of myelin integrity and axonal architecture (Alexander et al., 2007; O'Donnell and Westin, 2011). Even in GM, postmortem validation studies have shown that orientation and distribution of myelin and axons significantly contribute to FA (Kroenke et al., 2007; Jespersen et al., 2012; Seehaus et al., 2015). Since our histology data did not contain myelin stains, it is not surprising that there is only a low to moderate correlation with cell composition observed relative to FA. It is likely that similar to FA, the biological factors of myelin and axons could also be a major contributor to RTOP which is not captured by our Nissl-stained cell bodies and could be the reason why our study found no significant contribution of cell composition on RTOP and should be investigated in future studies.

**Figure 8:**
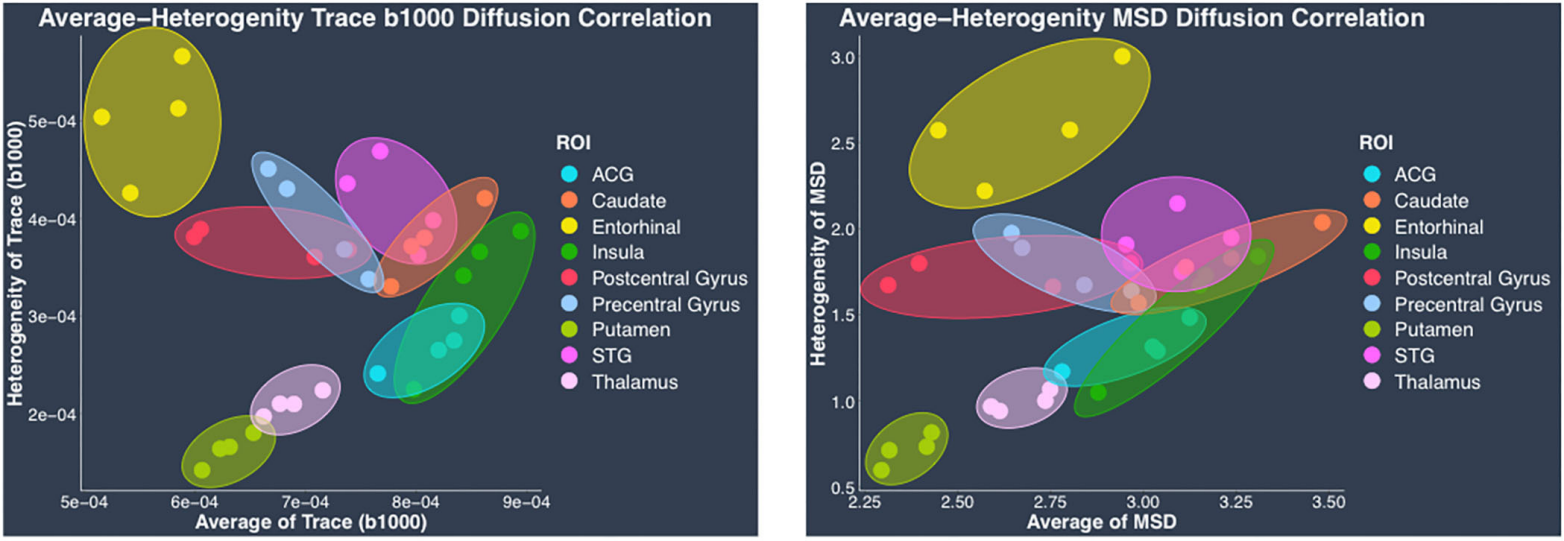
Regional average vs. heterogeneity of dMRI measures. In this figure, scatter plots of regional average vs. heterogeneity of dMRI measures: DTI-derived trace (left) and biexponential model-derived MSD (right), visually show clustering of each region from four monkeys. In each plot, individual points have been color coded to represent a region from the four monkeys. Clusters of each region from four monkeys have been delineated using color coded ellipses. The color coding for the regions is as follows: anterior cingulate gyrus (ACG) in sky blue, caudate in orange, entorhinal gyrus in yellow, insula in dark green, postcentral gyrus in red, precentral gyrus in light blue, putamen in light green, superior temporal gyrus (STG) in dark pink, and thalamus in light pink.

In contrast to the regional heterogeneity dMRI measures, the regional average dMRI measures estimated from both DTI and the biexponential model did not show significant correlation with the histological measure of regional average cell area density when all the regions (cortical and subcortical) were included together in the correlation analysis. However, separate analysis conducted in cortical and subcortical regions revealed opposite trends showing positive correlation between average trace, MSD, and average cell area density in cortical regions whereas negative correlation in subcortical regions. These opposing correlation trends observed between average trace, MSD, and average cell area density in cortical vs. subcortical regions, could explain the reason why we did not see any significant correlation when including all the regions (cortical and subcortical) together in the analysis. Future studies with data from more subcortical regions and perhaps myelin, dendrites, axonal, and cell type specific stains are needed to understand the reason behind such opposing correlation trends observed between the average dMRI and histology measures in cortical vs. subcortical regions. Nevertheless, our results show that regional average dMRI measures of trace and MSD are sensitive to the underlying biological feature of average cell area density. Additionally, the two-dimensional scatter plots of DTI-derived trace measure (hTrace vs. avgTrace) and biexponential-derived MSD measure (hMSD vs. avgMSD) (Figure 8), both showed that the same regions from all four monkeys visually clustered together.

Clustering of the same regions from all four monkeys demonstrates that average and heterogeneity dMRI measures together show regional specificity, suggesting sensitivity to microstructural tissue properties. It is important to note that in this study we only investigated the histological measure of cell composition, and it is possible that other biological features of myelin, axons, and dendrites are the likely contributors to the dMRI measures.

## Future implications

Investigation of the contribution of underlying cytoarchitecture to the dMRI measures, provided us with the much needed validation for dMRI measures of trace and MSD regarding their ability to capture changes in cell composition and organization in GM. Changes in cell density and cell size as well as position due to cell migration, have been observed during development and aging (Götz et al., 2016; Martínez-Pinilla et al., 2016; Nakafuku and Del Águila, 2020; d'Alessandro et al., 2021; Sikora et al., 2021). Non-invasive imaging measures of hTrace, hMSD along with average trace, and average MSD could be used as important tools in future longitudinal research studies investigating GM changes during normal development and aging. In addition, abnormalities in the cellular microstructure have been reported in relation to several developmental as well as degenerative disorders such as autism, schizophrenia, Alzheimer's, and Parkinson's (Arnold et al., 1991; Cho et al., 2016; Martínez-Pinilla et al., 2016; Giguère et al., 2018). Our measures of MSD and trace could thus prove extremely valuable biomarkers in the diagnosis and treatment monitoring of such disorders.

## Limitations

This study has a few limitations that must be acknowledged. We acknowledge that other biological factors such as synaptic changes (synaptogenesis, pruning) or changes in dendritic arborization, myelination, axonal orientation could also influence dMRI measures, which should be investigated in future work. In addition, our histological study used spaced sections forcing our analysis to be conducted on 2D sections within a region, leading to local discontinuities. In the future, methods such as CLARITY that allow conducting histological measurements in whole intact tissue followed by 3D imaging could be used for validation of dMRI-based microstructure estimates. Nevertheless, this study is an important first step toward histological validation of dMRI measures in GM in macaque.

## Conclusion

Based on the results of our study we conclude that dMRI measures can serve as imaging biomarkers of GM cellular structure and organization. Average and heterogeneity in dMRI measures of trace and MSD appear to be best suited to study the underlying cytoarchitecture. Direct quantitative comparisons conducted in this study between these dMRI measures and histological features of cytoarchitecture would provide an important guide to interpreting the results of studies using these dMRI model-derived measures. These dMRI measures of hTrace, hMSD, average trace and average MSD thus have the potential for use as non-invasive imaging biomarkers in studies that involve investigation of the changes in GM cytoarchitecture related to development and aging in healthy populations as well as abnormal cellular pathologies in clinical studies.

## Data availability statement

The raw data supporting the conclusions of this article will be made available by the authors, without undue reservation.

## Ethics statement

The animal study was reviewed and approved by Boston University Institutional Animal Care.

## Author contributions

MK and YR contributed to the conception and design of the study as well as supervised and provided inputs throughout the study. GP, BJ, and AK scanned the animals and acquired the imaging data. NM provided inputs related to neuroanatomy during the interpretation of results and drawing of the anatomical regions. DR and TM harvested the tissue samples upon sacrificing the same animals and conducted immunohistochemistry. SC-K contributed to the image analysis. MB digitized the stained histology slices, performed image and statistical analysis as well as wrote the article. All authors read the manuscript, contributed to the revision and approved the submitted version.

## Funding

We gratefully acknowledge the funding provided by following grants: RO1 AG042512 National Institute on Aging (PI: MK, NM, and DR); RF1 AG043640 (PI: DR). R01 MH111917 National Institute of Mental Health (PI: YR); R01 MH102377 National Institute of Mental Health (PI: MK); R01 MH112748 National Institute of Mental Health (PI: MK and NM); R01 MH119222 National Institute of Mental Health (PI: YR); K24 MH110807 National Institute of Mental Health (PI: MK).

## Conflict of interest

The authors declare that the research was conducted in the absence of any commercial or financial relationships that could be construed as a potential conflict of interest.

## Publisher's note

All claims expressed in this article are solely those of the authors and do not necessarily represent those of their affiliated organizations, or those of the publisher, the editors and the reviewers. Any product that may be evaluated in this article, or claim that may be made by its manufacturer, is not guaranteed or endorsed by the publisher.
